# The Combination of Morphological and Phylogenetic Evidence Reveals Four New *Gymnopus* Species and New Distribution

**DOI:** 10.3390/jof10100672

**Published:** 2024-09-27

**Authors:** Jia-Jun Hu, Yong-Lan Tuo, Zheng-Xiang Qi, Xue-Fei Li, Dong-Hua Jiang, Bo Zhang, Yu Li

**Affiliations:** 1 School of Life Science, Zhejiang Normal University, Jinhua 321004, China; hujjfungi@163.com (J.-J.H.); jdh@zjnu.cn (D.-H.J.); 2 Joint Laboratory of International Cooperation in Modern Agricultural Technology, Ministry of Education, Jilin Agricultural University, Changchun 130118, China; tuoyonglan66@163.com (Y.-L.T.); qzx7007@126.com (Z.-X.Q.); lixuefei2020@163.com (X.-F.L.); 3 Engineering Research Center of Edible and Medicinal Fungi, Ministry of Education, Jilin Agricultural University, Changchun 130118, China

**Keywords:** gymnopoid, new species, Omphalotaceae, habitat

## Abstract

The genus *Gymnopus* plays a significant role in ecological systems, with certain species holding potential as food or medicinal resources. However, the species diversity of *Gymnopus* in China remains unclear. In recent years, more than one thousand *Gymnopus* specimens have been collected across China. Thus, through the integration of ecological evidence, detailed morphological studies, and phylogenetic analysis using a multiloci dataset of ITS + nLSU + *tef1-ɑ*, four new species—*Gymnopus longistipes*, *Gymnopus striatipileatus*, *Gymnopus viridiscus*, and *Gymnopus spadiceus*—have been differentiated from known species. *Gymnopus similis* has been newly documented from Jiangxi Province, China. Detailed descriptions and vivid illustrations have been provided based on the newly collected specimens, along with comparisons to closely related species. Additionally, a key to the reported species of *Gymnopus* s.l. from East China has been included.

## 1. Introduction

The genus *Gymnopus* (Pers.) Roussel holds high economic and ecological value [1]. For example, species belonging to the *Gymnopus dryophilus* (Bull.) Murrill complex are hunted and consumed as food resources in China [2]. Additionally, *Gymnopus androsaceus* (L.) Della Magg. & Trassin. has been developed for medicinal purposes [3]. However, the species diversity of *Gymnopus* in China has been greatly overlooked.

Taxonomic research on *Gymnopus* has been ongoing for a significant period. Initially, the genus *Gymnopus* was considered a tribe within *Agaricus* L. [4]. Later, Fries transferred these species to trib. *Collybia* Fr., as proposed by Fries [5]. Subsequently, Staude [6] established the genus *Collybia* (Fr.) Staude. Researchers followed the opinions of Fries and Staude until Singer divided the genus *Collybia* into nine sections in his book, *The Agaricales in Modern Taxonomy* [7,8,9,10], which became the mainstream view. Although some researchers proposed their own perspectives on the taxonomic systems [11,12], Singer’s classification remained widely accepted. However, with the advancement of taxonomic studies in *Collybia*, Halling [13] and Antonín and Noordeloos [14,15] identified problematic taxonomy within the genus *Collybia*. As a result, Antonín et al. [16] proposed the establishment of the genus *Gymnopus*, transferring the species into it and leaving only four species within *Collybia*.

The application of molecular studies in the genus *Gymnopus* has sparked debates regarding the boundaries between *Gymnopus* and allied genera [17,18,19]. The concept of the genus *Gymnopus* is changing, as evidenced by recent changes. For instance, Noordeloos and Antonín [20] combined sect. *Androsacei* (Kühner) Antonín & Noordel. into *Gymnopus*. In a more recent development, Oliveira et al. [21] elevated sect. *Perforantia* (Singer) R.H. Petersen to the genus rank, naming it *Paragymnopus* J.S. Oliveira, and transferred sect. *Vestipedes* (Fr.) Antonín, Halling & Noordel. to *Marasmiellus* Murrill (later confirmed to be a synonym of *Collybiopsis* (J. Schröt.) Earle [22]), thereby establishing a distinct boundary between *Gymnopus* and *Collybiopsis*. However, this also leaves the multiloci phylogeny of *Gymnopus* and *Collybiopsis* [23]. Through continued research, the now genus *Gymnopus* has become delimited as a group, which is characterized by a collybioid appearance, rarely tricholomatoid or marasmioid; free to adnate and usually crowded lamellae; an insititious stipe, or not; a white spore print; basidiospores ellipsoid to short-oblong, inamyloid; cheilocystidia usually present and varied; a cutis or ixocutis pileipellis with radially arranged cylindrical hyphae or interwoven more akin to a trichoderm or ixotrichoderm, made up of irregular coralloid terminal elements (“*Dryophila* structures”), often incrusted, diverticulate hyphal elements, mixed with broom cells and coralloid hyphae; and clamp connections present in all tissues [21,24].

Furthermore, several infrageneric taxonomic issues remain unresolved. Within the genus *Gymnopus*, the foetid species was previously classified in sect. *Vestipedes* due to their similar tomentose stipes. This classification was later elevated to section rank due to the indistinct odour [24]. However, some foetid species still reside in sect. *Vestipedes*. Additionally, there are species that do not align well with the sectional characteristics. For example, *Gymnopus earleae* Murrill can be easily distinguished from other species in sect. *Levipedes* by its absence or inconspicuous cheilocystidia and the non-diverticulate or coralloid pileipellis, which are considered key characteristics of this section. The dense stipe vesture, inconspicuous cheilocystidia, and presence of caulocystidia allow *Gymnopus kauffmanii* (Halling) Halling to be differentiated from sect. *Levipedes*.

To date, approximately 300 *Gymnopus* species have been described globally, with North America, Europe and Asia reporting the highest numbers of species, accounting for 39.14%, 18.75% and 14.14% of the total, respectively. In contrast, Oceania and Africa have reported the fewest species.

The taxonomic studies of *Gymnopus* in China are comparatively less advanced than those conducted abroad. The earliest recorded instance of *Collybia* (*Gymnopus*) could be track back to the book *Fungi of China* [25]. Subsequently, Liu et al. [26] described the first new species of *Collybia* from China, *Collybia citrina* B. Liu, Rong & H.S. Jin. Since then, several new species, previously undocumented in China and new to science, have been reported [23,27,28,29,30,31,32,33,34]. However, research on *Gymnopus* is uneven, with Northeast China and South China reporting the highest number of species. Nonetheless, a comprehensive taxonomic study of *Gymnopus* in China is still lacking.

This paper aims to increase the species diversity of *Gymnopus* in China based on the combined ecology, morphology, and phylogenetic evidence. Furthermore, it aims to report the new distribution area of *Gymnopus similis* Antonín, Ryoo, and Ka in China.

## 2. Materials and Methods

### 2.1. Sampling and Morphological Study

Specimens were collected using the random sampling method and photographed in situ. The sizes of the basidiomata were measured when fresh. Following the examination and recording of the macroscopic characteristics, the specimens were dried in an electric drier at approximately 45 °C. Then the dried specimens were stored in self-zip plastic bag with colour silica gel.

The macroscopic characteristics were documented using filed notes and photographs. Additionally, colour descriptions were conducted based on the *Flora of British Fungi: Colour Identification Chart* [35]. Ethanol 94% was used to rehydrate the dried specimen before microscopic examination. Subsequently, they were examined under a Zeiss Axio lab. A1 microscope at magnifications up to 1000×. During the examination, 3% potassium hydroxide (3% KOH), 1% Congo red solution, or Melzer’s reagent [36], were employed as floating agents. All measurements were taken from sections mounted in 1% Congo red. For each specimen, characteristics such as basidiospores, basidia, cheilocystidia, or width of pileipellis elements, etc., were measured 40 times from at least two different basidiomata. The measurements were recorded as length × width (L × W). Of the obtained measurements, 5% were considered outliers and were excluded from each extreme end of the range and then given in parentheses. The basidiospores’ variation in the ratio of L to W among the studied specimens was denoted as Q. Qm represented the average Q value of all the basidiospores ± the standard deviation. The examined specimens are deposited in the herbarium of Jilin Agricultural University (HMJAU). 

### 2.2. DNA Extraction, PCR Amplification, and Sequencing

The total DNA was extracted from dried specimens or biomaterials using the NuClean Plant Genomic DNA Kit (Kangwei Century Biotechnology Company Ltd., Beijing, China), following the manufacturer’s instructions. For each specimen, about 10 mg starting material was used for DNA extraction. Phylogenetic analysis utilized sequences from the internal transcribed spacer (ITS) region, nuclear large ribosomal subunits (nLSU), and translation elongation factor 1-α (*tef1-ɑ*). The primer pairs ITS1-F/ITS4-B [37], LR0R/LR7 or LR5 [38,39], and tef1f/tef1r [40] or 983f/1953r were employed to amplify the ITS, nLSU, and *tef1-ɑ* regions, respectively. Each PCR reaction (25 μL) consisted of 12.5 μL 2 × EasyTaq PCR SuperMix (TransGen Biotech Co., Ltd., Beijing, China), 1 μL of each 10 μM primer, 2 μL DNA solution, and 8.5 μL dd H_2_O [23,33]. The reaction programs followed those described by Coimbra et al. [41] for ITS, Ryoo et al. [42] for nLSU, and Xu et al. [43,44] for *tef1-ɑ*. The PCR products were visualized under UV light after electrophoresis on 1.2% agarose gels stained with ethidium bromide. Subsequently, the purified PCR products were sent to Sangon Biotech Limited Company (Shanghai, China) for sequencing using the Sanger method through Thermo Fisher TM 3730XL Analyzer Applied Biosystems (Waltham, MA, USA). For newly described species, two or more collections were sequenced for their ITS, nLSU, and *tef1-ɑ* sequences. The newly obtained sequences were deposited in GenBank (http://www.ncbi.nlm.nih.gov/genbank; Table 1).

### 2.3. Data Analysis

Representatives of published sequences from closely related species to the new Chinese species (i.e., >97% similarity in BLASTn results) were included (Table 1). A combined dataset of ITS, nLSU, and *tef-1ɑ* sequences included sequences from this study, comprising 49 sequences obtained from the type species. Furthermore, some *Collybiopsis* (J. Schröt.) Earle and *Marasmiellus* Murrill species were selected for phylogenetic analysis due to the unclear of the boundaries between these genera and the taxonomic system changes recently. Additionally, species belonging to *Marasmius* Fr. were selected as outgroups [45].

In the dataset, each gene region was aligned using either Clustal X [46] or MAFFT 7.490 [47] and subsequently manually checked in BioEdit 7.0.5.3 [48]. The alignments of the ITS, nLSU, and *tef1-ɑ* sequences were then combined using Phylosuite 1.2.2 [49]. The partition homogeneity test (PHT) [50] was performed on the multigene dataset using PAUP 4.0b10 [51] with 1000 homogeneity replicates. The best-fit evolutionary model was estimated using ModelFinder [52]. Bayesian inference (BI) algorithms were employed to conduct the phylogenetic analysis, utilizing MrBayes 3.2.6 with a general time-reversible DNA substitution model and gamma distribution rate variation across the sites [53]. Four Markov chains were run for two independent runs, starting from random trees, until the split deviation frequency value was less than 0.01. Trees were sampled every 100 generations, with the first 25% of sampled trees discarded as burn-in. The remaining trees were used to construct a 50% majority consensus tree and calculate the Bayesian posterior probabilities (PP). Maximum likelihood (ML) analysis was performed using RaxmlGUI 2.0.10 [54] with 1000 bootstrap (BS) replicates to search for the optimal topology. The resulting trees were visualized using FigTree 1.4.3.

## 3. Results

### 3.1. Phylogenetic Analysis

**Figure 1:**
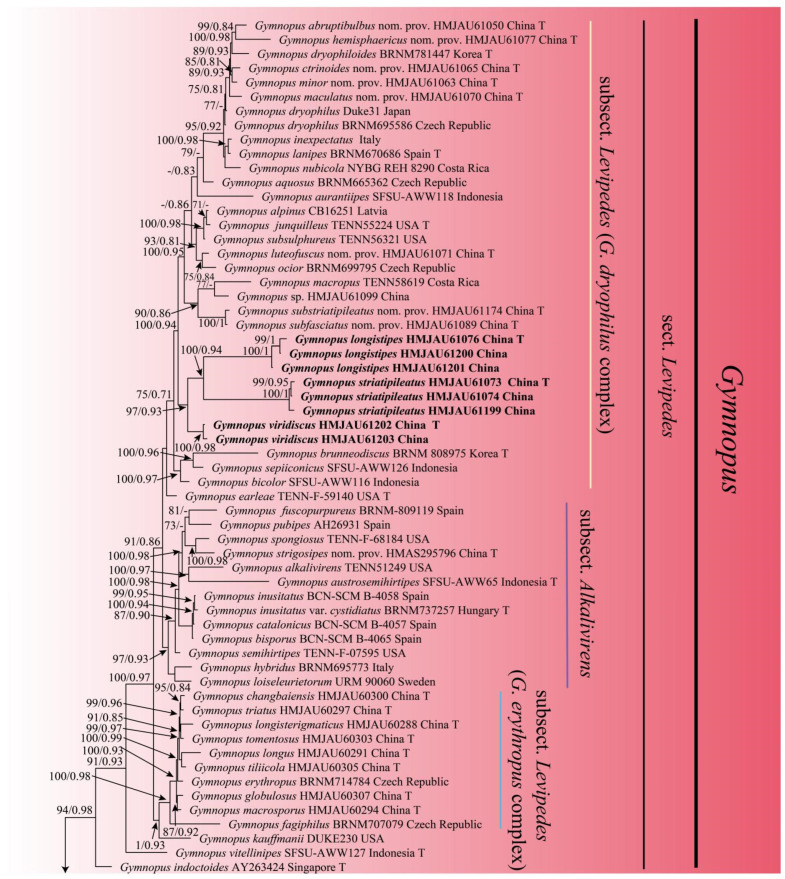

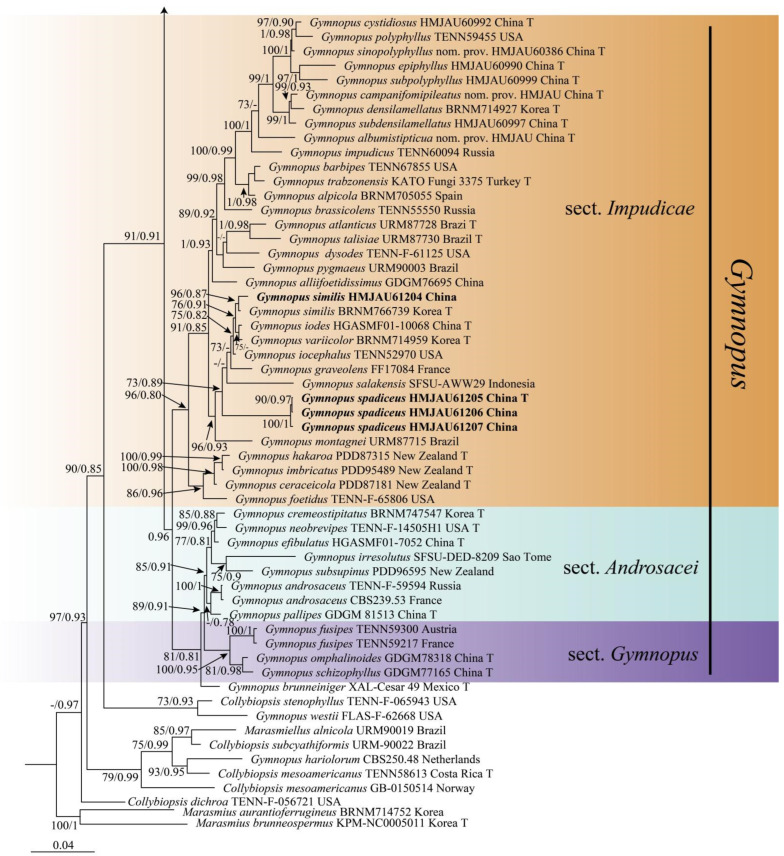
Maximum likelihood analysis generated from the combined ITS, nLSU, and *tef1-ɑ* dataset. Bootstraps values (BS) ≥ 70% from ML analysis and Bayesian posterior probabilities (PP) ≥ 0.80 are shown on the branches. Newly sequenced collections are indicated in bold, and the type specimens are denoted by (T).

In the combined dataset of ITS, nLSU, and *tef1-ɑ*, a total of 211 sequences were included from 121 different collections, with 115 sequences for ITS, 82 sequences for nLSU, and 14 sequences for *tef1-ɑ*. After trimming, the combined dataset contained 3237 characters, including gaps. Out of these sequences, 32 were newly obtained in the present study, with 12 sequences for ITS, 12 sequences for nLSU, and 8 for *tef1-ɑ*. The best model selected for the Bayesian inference (BI) analysis using ModelFinder in Phylosuite 1.2.2 was GTR+F+I+G4, while the ML analysis used the GTRGAMMA model. The Bayesian analysis was run for four million generations using MrBayes 3.2.6 in Phylosuite 1.2.2, resulting in an average standard deviation of split frequencies of 0.006578. The same dataset and alignment were also analyzed using the ML method in RaxmlGUI 2.0.10. Both the phylogenetic analyses yielded a congruent topology, which has been depicted in Figure 1.

The phylogeny inferred from the combined dataset showed that the *Gymnopus* split into four well-supported clades, representing sect. *Gymnopus*, sect. *Levipedes*, sect. *Androsacei,* and sect. *Impudicae*. Our newly achieved sequences formed four new well-supported lineages that were nested in sect. *Levipedes* and sect. *Impudicae* (Figure 1).

### 3.2. Taxonomy

#### 3.2.1. New Species

**Figure 2:**
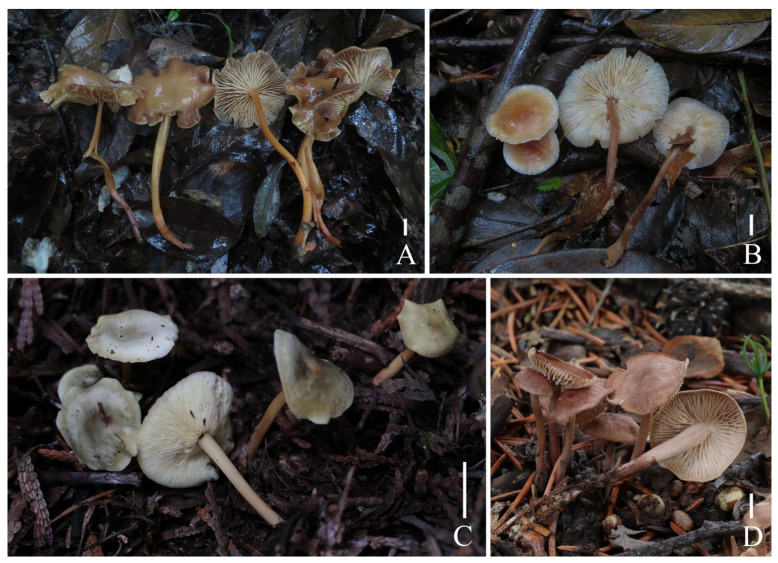
Habitat of *Gymnopus* species from China. (**A**) *Gymnopus longistipes*; (**B**) *Gymnopus striatipileatus*; (**C**) *Gymnopus viridiscus*; (**D**) *Gymnopus spadiceus*. Bars: 1 cm (**A**–**D**).

**Figure 3:**
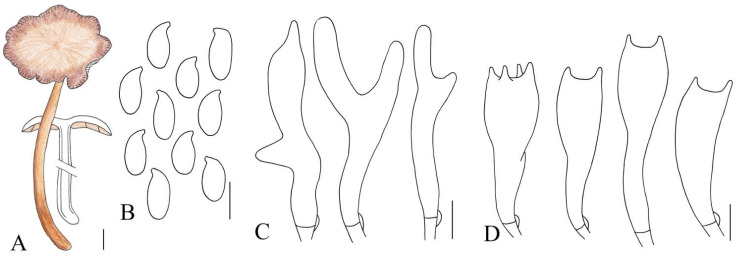
Illustration of *Gymnopus longistipes* (HMJAU 61076) morphological features. (**A**) Basidiomata; (**B**) Basidiospores; (**C**) Pileipellis elements; (**D**) Basidia. Bars: 1 cm (**A**); 10 μm (**C**); 5 μm (**B**,**D**).

***Gymnopus longistipes*** J.J. Hu, B. Zhang & Y. Li, **sp. nov.** (Figure 2A and Figure 3).

Fungal Name: FN 571752.

**Etymology:** refers to the long stipe of this species.

**Diagnosis** [English]: This species differs from closely related species due to large basidiomata, irregular pileus that are usually reddish brown, paler at disc, smooth, yellow to yellowish stipe, branched or coralloid pileipellis, absence of cheilocystidia and caulocystidia, and bigger basidiospores.

**Type:** China. Jiangxi Province: Ji’an City, Jinggangshan City, Jinggangshan Mountain, 6 May 2019, Jia-Jun Hu, Bo Zhang, and Dai Dan, HMJAU 61076 (**holotype** HMJAU 61076).

Basidiomata medium. Pileus 5.1–6.9 cm in diameter, convex, slightly depressed occasionally, yellowish brown to sepia, smooth, hygrophanous; margin entire, wavy, sepia to dark brown, striped, hygrophanous. Context thin, fresh, odourless. Stipe 7.1–11.0 × 0.4–1.2 cm, central, cylindrical, yellowish brown to reddish brown, smooth, striped, fistulose, fibrous. Lamellae adnate to adnexed, close, yellowish brown to light brown, unequal. Occurrence in leaf-litter.

Basidiospores (5.2)5.8–6.7(7.0) × (2.7)2.8–3.1(3.2) µm [Q = (1.73)1.93–2.23(2.33), Qm = 2.05 ± 0.14], elliptic, hyaline, smooth, inamyloid, thin-walled. Basidia (18)19–25(30) × 6–7 µm, clavate, two- or four-spored, hyaline, smooth, thin-walled. Cheilocystidia and caulocystidia absent. Pileipellis a cutis, 7–15(17) µm wide, made up of irregular branched or coralloid hyphae, flattened, hyaline, smooth, thin-walled. Clamp connections present.

**Habit, habitat, and distribution:** Scattered to gregarious. Saprotrophic, with humicolous habitat, found in broad-leaved forests. So far, only known from Jiangxi Province, China.

**Other specimens examined:** China. Jiangxi Province: Ji’an City, Jinggangshan City, Jinggangshan Mountain, 10 May 2020, Bo Zhang, and Dai Dan, HMJAU 61200; Jiangxi Province: Pingxiang City, Wugong Mountain, 13 May 2023, Jia-Jun Hu, and Dai Dan, HMJAU 61201.

**Note:** This species is characterized by large basidiomata, irregular pileus that are usually reddish brown, paler at disc, smooth, yellow to yellowish stipe, branched or coralloid pileipellis, absence of cheilocystidia and caulocystidia.

*Gymnopus longistipes* shares similarities with *Gymnopus aurantiipes* (Corner) A.W. Wilson, Desjardin & E. Horak, such as the yellow to orange basidiomata and long stipes. However, *G. longistipes* differs from *G. aurantiipes* by the close, but not crowded lamellae, not pruinose stipe upon drying, absence of cheilocystidia, and smaller basidiospores [55]. *Gymnopus longistipes* is a sister to *Gymnopus striatipileatus*, according to our phylogenetic analysis. However, *G. longistipes* can be distinguished from *G. striatipileatus* through the wavy margin of pileus, longer stipe, and absence of cheilocystidia.

**Figure 4:**
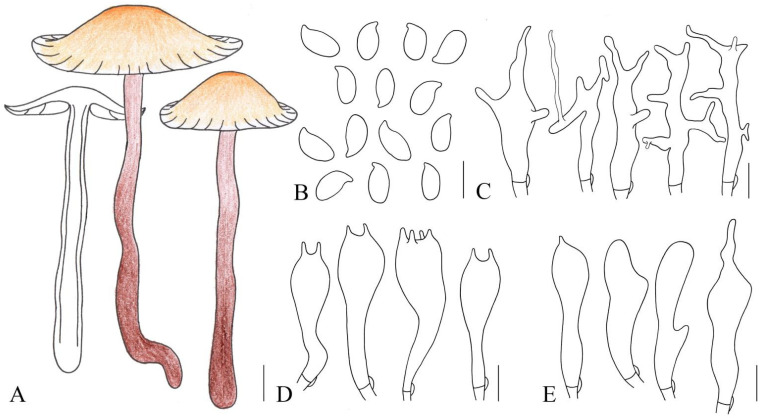
Illustration of *Gymnopus striatipileatus* (HMJAU 61073) morphological features. (**A**) Basidiomata; (**B**) Basidiospores; (**C**) Pileipellis elements; (**D**) Basidia; (**E**) Cheilocystidia. Bars: 1 cm (**A**); 10 μm (**C**); 5 μm (**B**,**D**,**E**).

***Gymnopus striatipileatus*** J.J. Hu, B. Zhang & Y. Li, **sp. nov.** (Figure 2B and Figure 4).

Fungal Name: FN 571371.

**Etymology:** refers to the striate pileus margin.

**Diagnosis** [English]: This species is differentiated from other species due to convex pileus that is brown at disc, light brown outwards, striped, cylindrical, brown to reddish brown stipe, irregular cheilocystidia with an umbo at apex, branched or coralloid pileipellis usually flattened, diverticulated, or with a long and slim branch, and smaller basidiospores.

**Type:** China. Jiangxi Province: Ji’an City, Jinggangshan City, Jinggangshan Mountain, 6 May 2019, Jia-Jun Hu, Bo Zhang, and Dai Dan, HMJAU 61073 (**holotype** HMJAU 61073).

Basidiomata small to medium. Pileus 1.5–5.2 cm in diameter, convex, brown to dark brown, paler outwards, yellow to light brown, smooth, glabrous, hygrophanous; margin entire, yellowish white to light brown, hygrophanous, striped. Context light brown, thin, fresh, odourless. Stipe 4.2–9.5 × 0.1–0.3 cm, central, cylindrical, yellowish white to light reddish brown, fistulose, fibrous. Lamellae adnexed, close, yellowish brown to light reddish brown, unequal. Occurrence in leaf-litter.

Basidiospores 5.0–6.0(6.1) × (2.4)2.6–3.0 µm [Q = (1.58)1.67–2.08(2.77), Qm = 1.84 ± 0.15], elliptic, hyaline, smooth, inamyloid, thin-walled. Basidia (12)14–24(26) × 4–6 µm, clavate, two- or four-spored, hyaline, smooth, thin-walled. Cheilocystidia abundant, 15–25 × 4–6(7) µm, irregularly clavate, with an umbo at apex, hyaline, smooth, thin-walled. Pileipellis a cutis, (5)6–12(13) µm wide, made up of irregular branched or coralloid hyphae, diverticulate, flatten, hyaline, smooth, thin-walled. Clamp connections present.

**Habit, habitat, and distribution:** Scattered to gregarious. Saprotrophic, with humicolous habitat, found in broad-leaved forests. So far, only known from Jiangxi Province, China.

**Other specimen examined:** China. Jiangxi Province: Ji’an City, Jinggangshan City, Jinggangshan Mountain, 3 May 2021, Bo Zhang, and Dai Dan, HMJAU 61074; Jiangxi Province: Ji’an City, Jinggangshan City, Jinggangshan Mountain, 20 May 2022, Bo Zhang, and Dai Dan, HMJAU 61199.

**Note:** This species is characterized by its convex pileus that is brown at the disc, turning light brown outwards, striped, cylindrical, brown to reddish brown stipe, irregular cheilocystidia with an umbo at the apex, branched or coralloid pileipellis that is usually flattened, diverticulate, or having long and slim branches, and smaller basidiospores.

This species bears a resemblance to *Gymnopus erythropus* (Pers.) Antonín, Halling & Noordel. due to its brown to reddish brown stipe. However, it can be differentiated from *G. erythropus* due to its smaller basidiospores and a pileipellis that is typically branched or coralloid, often flattened, diverticulate, or with long and slender branches [23,24,56]. *Gymnopus striatipileatus* differentiate from *Gymnopus globulosus* J.J. Hu, Y.L. Tuo, B. Zhang & Yu Li due to the habitat and coralloid pileipellis. [23].

**Figure 5:**
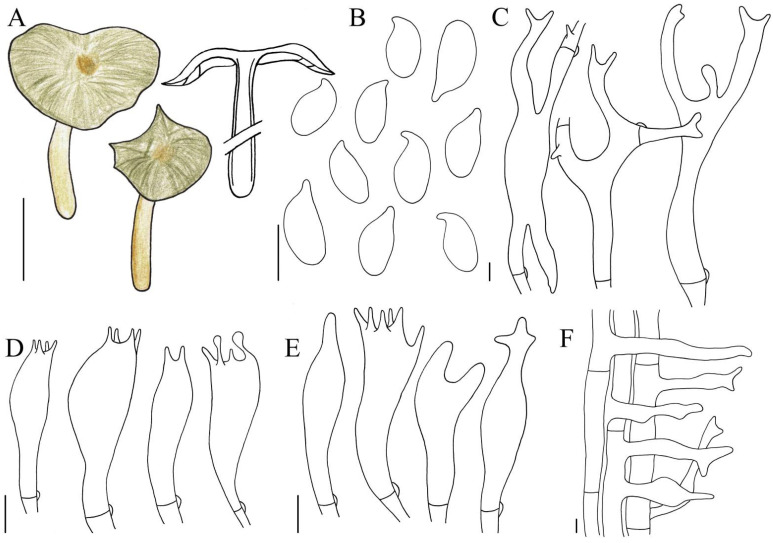
Illustration of *Gymnopus viridiscus* (HMJAU 61202) morphological features. (**A**) Basidiomata; (**B**) Basidiospores; (**C**) Pileipellis elements; (**D**) Basidia; (**E**) Cheilocystidia; (**F**) Caulocystidia. Bars: 1 cm (**A**); 5 μm (**B**–**F**).

***Gymnopus viridiscus*** J.J. Hu, Y.L. Tuo, B. Zhang & Y. Li, **sp. nov. (**Figure 2C and Figure 5).

Fungal Name: FN 571913.

**Etymology:** refers to the green pileus of the species.

**Diagnosis** [English]: This species can be differentiated from other species through its arising in early spring, its green pileus, *Dryophila*-structured but inflated pileipellis, presence of caulocystidia, and non-finger-like cheilocystidia.

**Type:** China. Zhejiang Province: Hangzhou City, Lin’an City, Tianmu Mountain, Longfengjian, 119.44 E, 30.34 N, 31 May 2021, Jin-Bao Pu, HMJAU 61202 (**holotype** HMJAU 61202).

Basidiomata small. Pileus 1.7–1.9 cm diameter, convex to applanate, depressed when mature, grayish green at disc, with yellow tone sometimes, grayish white outwards, smooth, hygrophanous; margin entire, wavy, striped, yellowish green, grayish green, or white. Context thin, white, odourless. Stipe 2.2–3.2 × 0.2–0.3 cm, central, cylindrical, fresh-coloured to light greenish yellow, darker at base, smooth, covered with furfuraceous or tomentose, fistulose, fibrous. Lamellae closed, white to light yellow, unequal. Occurrence in soil.

Basidiospores (5.1)5.2–6.2(6.3) × (2.5)2.8–3.3 μm [Q = (1.6)1.7–2.2, Qm = 2.0 ± 0.1], elliptic, hyaline, smooth, inamyloid, thin-walled. Basidia 16–24(26) μm, clavate, four-spored, occasionally two-spored, hyaline, thin-walled. Cheilocystidia scattered, clavate, bifurcated, finger-like, or with an umbo at apex, hyaline, thin-walled. Caulocystidia (30)50–85(110) × 4–6(7) μm, clavate, or bifurcated at apex, scattered, hyaline, thin-walled. Pleurocystidia absent. Pileipellis a cutis, (4)5–10(12) µm wide, made up of irregular branched or coralloid hyphae, inflated, hyaline, smooth, thin-walled. Clamp connections present.

**Habit, habitat, and distribution:** Scattered to gregarious. Saprotrophic, with terrestrial habitat, found in broad-leaved and coniferous mixed forests. So far, only known from Zhejiang and Anhui Province, China.

**Other specimen examined:** China. Anhui Province, Lu’an City, Jinzhai County, Tianma National Nature Reserve, 115.77 E, 31.16 N, 16 April 2023, Yong-Lan Tuo, HMJAU 61203.

**Note:** This species is characteristic of arising in early spring, its green pileus, *Dryophila*-structured but inflated pileipellis, presence of caulocystidia, and non-finger-like cheilocystidia.

This species is closely related to *Gymnopus aurantiipes* (Corner) A.W. Wilson, Desjardin & E. Horak, *Gymnopus fagiphilus* Antonín, Halling & Noordel., *Gymnopus indoctoides* A.W. Wilson, Desjardin & E. Horak, *Gymnopus inexpectatus* Consiglio, Vizzini, Antonín & Contu, *Gymnopus kauffmanii* (Halling) Halling, *Gymnopus macropus* Halling, *Gymnopus mucubajiensis* (Dennis) Halling, *Gymnopus nubicola* Halling, and *Gymnopus vitellinipes* A.W. Wilson, Desjardin & E. Horak, due to the presence of caulocystidia. However, G. *viridiscus* differs from these species in terms of its small basidiomata and grayish green pileus [24].

**Figure 6:**
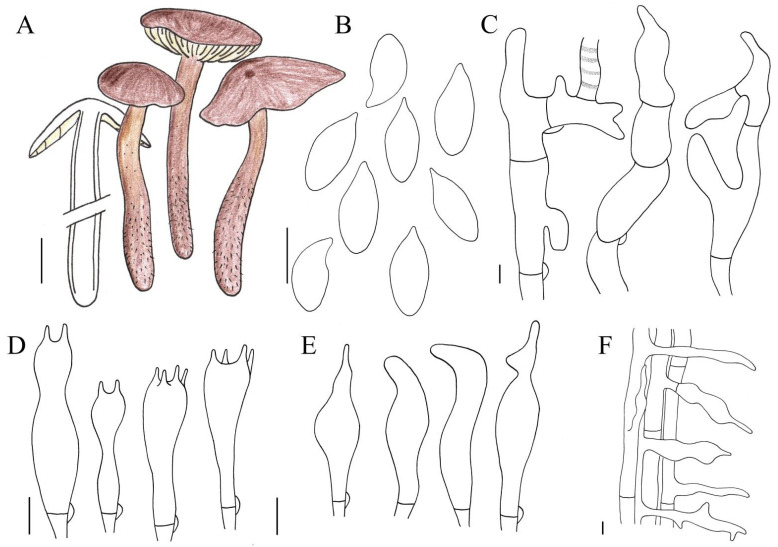
Illustration of *Gymnopus spadiceus* (HMJAU 61205) morphological features. (**A**) Basidiomata; (**B**) Basidiospores; (**C**) Pileipellis elements; (**D**) Basidia; (**E**) Cheilocystidia; (**F**) Caulocystidia. Bars: 1 cm (**A**); 10 μm (**F**); 5 μm (**B**–**E**).

***Gymnopus* *spadiceus*** J.J. Hu, B. Zhang & Y. Li, **sp. nov.** (Figure 2D and Figure 6).

Fungal Name: FN 571914.

**Etymology:** refers to the tan basidiomata of this species.

**Diagnosis** [English]: This species differs from other species due to its small basidiomata and branched pileipellis (weakly *Dryophila*-structured), grows on the leave litter of *Picea koraiensis* Nakai.

**Type:** China. Jilin Province: Changchun City, Jingyue District, campus of Jilin Agriculture University, 125.42 E, 43.82 N, 30 June 202, Jia-Jun Hu, HMJAU 61205 (**holotype** HMJAU 61205).

Basidiomata small. Pileus 0.3–1.8 cm in diameter, hemispherical to convex, or reflex sometimes, depressed at disc occasionally, smooth, glabrous, striped to half, tan, deep colour at disc; margin entire, involute to reflex, paler than center. Context thin, with strong smell reminiscent of rotten cabbage. Stipe 1.5–4.7 × 0.1–0.4 cm, central, cylindrical or tapering downwards sometimes, tan, paler at apex, tomentose. Lamellae middle, adnate to adnexed, paler than pileus, unequal. Occurrence in leaf-litter.

Basidiospores (5.2)6.0–7.4(8.0) × (2.6)2.8–3.4 μm [Q = (1.7)1.9–2.4, Qm = 2.2 ± 0.2], elliptic, hyaline, smooth, inamyloid, thin-walled. Basidia (17)18–31 μm, clavate, two- or four-spored, hyaline, thin-walled. Cheilocystidia scattered, (16)17–31 μm, clavate, with an umbo at apex, hyaline, thin-walled. Caulocystidia abundant, (30)35–85 × 3–12 μm, clavate, branched or with an umbo at apex sometimes, hyaline, thin-walled. Pleurocystidia absent. Pileipellis a cutis, (4)5–10(12) µm wide, made up of irregular branched or coralloid hyphae (weakly *Dryophila*-structured), inflated, incrusted sometimes, hyaline, smooth, thin-walled. Clamp connections present.

**Habit, habitat, and distribution:** Gregarious. Saprotrophic, with humicolous habitat, found in leaf litter of *P. koraiensis*. So far, only known from Jilin Province, China.

**Other specimen examined:** China. Jilin Province: Changchun City, Jingyue District, campus of Jilin Agricultural University, 125.42 E, 43.82 N, 17 July 2023, Jia-Jun Hu, HMJAU 61206 (Collection no.: Hu J.J. 601); Changchun City, Jingyue District, campus of Jilin Agricultural University, 125.42 E, 43.82 N, 17 July 2023, Jia-Jun Hu, HMJAU 61207 (Collection no.: Hu J.J. 604).

**Note:** This species is characterized through its small basidiomata, branched pileipellis (weakly *Dryophila*-structured), grows in the leaf litter of *Picea koraiensis* Nakai.

The species *Gymnopus spadiceus* is often confused with *Gymnopus similis* Antonín, Ryoo & Ka due to their similar appearances. However, *G. spadiceus* can be distinguished from *G. similis* due to its gymnopoid appearance, smaller basidiomata, weakly *Dryophila*-structured pileipellis, and clavate cheilocystidia [42]. In China, *Gymnopus iodes* J.P. Li, Chang Tian Li, Chun Y. Deng & Yu Li can also be confused with *G. spadiceus*; however, *G. iodes* can be separated due to their marasmioid appearance, shape of cheilocystidia, and caulocystidia [31].

#### 3.2.2. New Record of Jiangxi Province, China

***Gymnopus similis*** Antonín, Ryoo & Ka

**Specimen examined:** China. Jiangxi Province: Pingxiang City, Lvxi County, Wugong Mountain, Jia-Jun Hu, Zheng-Xiang Qi, and Dan Dai, 5 July 2023, HMJAU 61204.

**Note:** This species was originally described from South Korea, and first recorded from Zhejiang Province, China. This time, a similar specimen was collected from Jiangxi Province. A combination of morphological and molecular study confirmed it was *G. similis*. Before this time, it was never recorded in Jiangxi Province, China.

**Key to the reported *Gymnopus*** **s.l. and related species from East China**

1 Basidiospores amyloid
*Rhodocollybia butyracea*
1 Basidiospores inamyloid22 Basidiomata with foetid smell32 Basidiomata without foetid smell63 Basidiomata white, small
*Gymnopus alliifoetidissimus*
3 Basidiomata not white, small to medium44 Basidiomata marasmioid, covered with tomentose at stipe
*Gymnopus similis*
4 Basidiomata gymnopoid, covered with pruinose at stipe55 Basidiomata small, arose from coniferous forest
*Gymnopus spadiceus*
5 Basidiomata small to medium, arose on the ground of broad-leaved forest
*Gymnopus dysodes*
6 Stipe smooth76 Stipe usually covered with tomentose or pruinose147 Basidiomata marasmioid
*Gymnopus androsaceus*
7 Basidiomata not marasmioid88 Pileipellis green in KOH
*Gymnopus fuscopurpureus*
8 Pileipellis not green in KOH99 Stipe red109 Stipe not red, usually happens in early spring or late autumn1110 Pileus red, grows on ground
*Gymnopus erythropus*
10 Pileus yellow to lemon yellow, grows on leaves
*Gymnopus subsulphureus*
11 Cystidia absent
*Gymnopus longistipes*
11 Cystidia present1212 Caulocystidia present and massive1312 Caulocystidia absent or rare
*Gymnopus dryophilus*
13 Basidiomata marasmioid, stipe red
*Gymnopus striatipileatus*
13 Basidiomata gymnopoid, stipe fresh to light greenish yellow
*Gymnopus viridiscus*
14 Stipe fusiformis, pileipellis not Dryophila-structure
*Gymnopus fusipes*
14 Stipe usually cylindrical, pileipellis usually Dryophila-structure1515 Basidiomata small, marasmioid1615 Basidiomata small to medium, usually gymnopoid1716 Basidiomata small, grows on leaves
*Gymnopus hirtellus*
16 Basidiomata, medium to large, grows on ground
*Collybiopsis*
*peronat*
*a*
17 Cystidia absent1817 Cystidia present1918 Hyphae blue in KOH
*Gymnopus iocephalus*
18 Hyphae not blue in KOH
*Gymnopus castaneus*
19 Stipe with white mycelioid at the base2019 Stipe without white mycelioid at the base2120 Caulocystidia absent
*Collybiopsis*
*biformis*
20 Caulocystidia present
*Collybiopsis*
*confluens*
21 Pileus covered with obvious stripe
*Collybiopsis*
*polygramm*
*a*
21 Pileus without obvious stripe
*Collybiopsis*
*luxurians*


## 4. Discussion

This study presents the detailed description of four new species belonging to sect. *Levipedes* and sect. *Impudicae*, which were collected from East and Northeast China. These new species are well-supported by a combination of ecological characteristics, morphological evidence, and phylogenetic analysis. Furthermore, the newly recognized and delimited species belonging to sect. *Levipedes* were found to occur in early spring in broad-leaved forests.

### 4.1. Morphological Characteristics in Genus and Species Delimitation

In taxonomy under genus level, the type of pileipellis is commonly regarded as a significant feature. Antonín et al. [16] classified *Gymnopus* based on a combination of pileipellis structure, basidiomata shape, and odour, dividing the genus into four sections. The criteria for genus and species delineation have been subject to ongoing evolution. The marasmioid appearance and presence of broom cells in the pileipellis were previously considered distinguishing features of sect. *Androsacei*. However, this led to the recognition of the polyphyletic nature of sect. *Androsacei*, which was subsequently resolved through the use of Melinda’s reagent [57].

In the *Gymnopus dryophilus* complex, similar challenges have been encountered. Initially, it was believed that the size of the basidiospores, pileus colour, and shape of cheilocystidia were key features for distinguishing species within the *G. dryophilus* complex [58,59]. However, a combination of morphological analysis and molecular studies conducted by Antonín et al. [56] revealed that the colour of lamellae and shape of cheilocystidia play a significant role in species differentiation. Additionally, the foetid smell was considered a significant characteristic of sect. *Impudicae*. However, there are species within this section that lack a distinct smell, and some species in sect. *Vestipedes* that have a strong smell. Furthermore, there are species that do not always conform to the characteristics of their respective sections, and some species exhibit variations. For example, *Gymnopus montagnei* (Berk.) Redhead lacks clearly defined lamellae and resembles a goblet. As a result, some researchers have classified it as a member of *Hypolyssus* Pers. or *Perona* Pers., but phylogenetic analysis confirms its membership in sect. *Impudicae* [33]. In the case of sect. *Levipedes*, the pileipellis is typically composed of flattened and radially arranged hyphae (*Dryophila*-structure). However, in a previous study, the pileipellis of *Gymnopus globulosus* J.J. Hu, Y.L. Tuo, B. Zhang & Yu Li was observed to have two layers. The lower layer exhibited the typical *Dryophila*-structure, while the terminal hyphae formed bulbous structures, which had not been previously observed in this section [23]. These examples shed light on the taxonomic challenges faced in understanding the diversity, characteristics, and limits of the genus *Gymnopus*. Although the name “*Gymnopus*” was proposed in 1801 by Persoon, our knowledge of its species diversity and taxonomic characteristics remains limited.

### 4.2. Phylogenetic Relationships within Gymnopus

Phylogenetic analyses of *Gymnopus* s.l. within taxonomic systems have been ongoing for approximately two decades. Researchers are striving to determine the monophyletic nature of *Gymnopus* [16,18,19,21,57]. However, to date, the relationships within and among *Gymnopus* species remain contentious.

The relationships within *Gymnopus* require in-depth analysis. *Gymnopus* comprises species with collybioid basidiomata, occasionally marasmioid, white spore prints, and typically smooth, inamyloid, and hyaline basidiospores [21,24]. Recently, Hu [57] conducted a comprehensive phylogenetic analysis on *Gymnopus* specimens from China, proposing a novel perspective. However, this new perspective presented challenges in accurately placing all species. For instance, most sect. *Impudicae* species exhibit distinct smells, matte stipes, and non-flattened pileipellis, while some species lack the foetid odor, cheilocystidia, or typical gilled structures. Sect. *Levipedes* species are characterized by a *Dryophila*-like pileipellis and easily identifiable cheilocystidia. Some species did not conform to typical clustering patterns. Therefore, comprehensive and systematic studies are needed for these groups.

The relationships within the genus *Gymnopus* are a subject of debate and contention. Section *Levipedes* has been subdivided into two subsections, subsect. *Levipedes* Antonín & Noordel. and subsect. *Alkalivirens* Antonín & Noordel., based on whether the pileus turns green in potassium hydroxide solution (KOH) [60]. Additionally, due to a lack of interest, *G. erythropus*/G. *fagiphilus* have often been grouped within subsect. *Levipedes*. However, previous studies suggest that G. *erythropus*/*G. fagiphilus* may have a more distant relationship with other species in subsect. *Levipedes*. Furthermore, our previous study of red stipe specimens from Northeast China also confirmed a polyphyletic relationship between these species [23]. Therefore, an in-depth investigation of *Gymnopus* is warranted.

## Figures and Tables

**Table 1:** Voucher/specimen numbers, country, and GenBank accession numbers of the specimens included in this study. Sequences produced in this study are in bold. The sequences from type specimens are marked as T.

Scientific Name	Voucher/Specimen Numbers	Country	GenBank Accession Numbers		
			ITS	nLSU	*tef1-ɑ*
*Collybiopsis mesoamericanus* (T)	TENN58613	Costa Rica	DQ450035	KY019632	
*C. stenophylla*	TENN-F-065943	USA	MN413331	MW396886	
*C. subcyathiformis*	URM-90022	Brazil	KY404983	KY404983	
*C. dichroa*	TENN-F-056721	USA	KY026654	KY026654	
*Gymnopus abruptibulbus* (T)	HMJAU61050	China	OQ597084		
*G. albumistipticus* (T)	HMJAU61032	China	OQ597081		
*G. alkalivirens*	TENN51249	USA	DQ450000		
*G. alliifoetidissimus*	GDGM76695	China	MT023344	MT017526	
*G. alpicola*	BRNM705055	Spain		MK278102	
*G. alpinus*	CB16251	Latvia	JX536168		JX536191
*G. androsaceus*	TENN-F-59594	Russia	KY026663	KY026663	
*G. androsaceus*	CBS239.53	France	MH857174	MH868713	
*G. aquosus*	BRNM665362	Czech Republic	JX536172		JX536192
*G. atlanticus* (T)	URM87728	Brazil	KT222654	KY302698	
*G. aurantiipes*	SFSU-AWW118	Indonesia	AY263432	AY639410	
*G. austrosemihirtipes* (T)	SFSU-AWW65	Indonesia	AY263422		
*G. barbipes*	TENN67855	USA	KJ416269	NG_059733	
*G. bicolor*	SFSU-AWW116	Indonesia	AY263423	AY639411	
*G. bisporus*	BCN-SCM-B-4065	Spain	JN247551	JN247555	
*G. brassicolens*	TENN55550	Russia	DQ449989		
*G. brunneiniger* (T)	XAL-Cesar 49	Mexico	MT232389	NG-075396	
*G. brunneodiscus* (T)	BRNM808975	Korea	MH589975	MH589991	
*G. campanifomipileatus* (T)	HMJAU61027	China	OQ597064	OQ594474	
*G. catalonicus*	BCN-SCM-B-4057	Spain	JN247552	JN247556	
*G. ceraceicola* (T)	PDD87181	New Zealand	KC248405		
*G. changbaiensis* (T)	HMJAU60300	China	OM030272	OM033387	
*G. cremeostipitatus* (T)	BRNM747547	Korea	KF251071	KF251091	
*G. ctrinoides* (T)	HMJAU61069	China	OQ597053	OQ594463	
*G. cystidiosus* (T)	HMJAU60992	China	ON259024	ON259036	
*G. densilamellatus* (T)	BRNM714927	Korea	KP336685	KP336694	
*G. dryophiloides* (T)	BRNM781447	Korea	MH589967	MH589985	
*G. dryophilus*	BRNM695586	Czech Republic	JX536143		JX536196
*G. dryophilus*	Duke31	Japan	DQ480099		
*G. dysodes*	TENN-F-61125	USA	KY026666		
*G. earleae* (T)	TENN-F-59140	USA	DQ449994	KY019634	
*G. efibulatus*	HGASMF01-7052	China	OM970865	OM970865	
*G. epiphyllus* (T)	HMJAU60990	China	ON259030	ON259038	
*G.erythropus*	BRNM714784	Czech Republic	JX536136		
*G. fagiphilus*	BRNM707079	Czech Republic	JX536129		
*G. foetidus*	TENN-F-65806	USA	KY026682	KY026682	
*G. fuscopurpureus*	BRNM-809119	Spain	MZ542559	MZ542563	
*G. fuscotramus*	GDGM26313	China	JF303730		
*G. fusipes*	TENN59300	Austria	AF505777		
*G. fusipes*	TENN59217	France	AY256710	AY256710	
*G. globulosus* (T)	HMJAU60307	China	OM030269	OM033406	
*G. graveolens*	FF17084	France	MH422573	MH422572	
*G. hakaroa* (T)	PDD87315	New Zealand	KC248410		
*G. hariolorum*	CBS250.48	Netherlands	MH856329	MH867883	
*G. hemisphaericus* (T)	HMJAU61077	China	OQ597057	OQ594467	
*G. hybridus*	BRNM695773	Italy	JX536177		
*G. imbricatus* (T)	PDD95489	New Zealand	KC248390		
*G. impudicus*	TENN60094	Russia	KJ416263		
*G.indoctoides* (T)	AY263424	Singapore	AY639419		
*G. inexpectatus*		Italy	EU622905	EU622906	
*G. inusitatus*	BCN-SCM-B-4058	Spain	JN247553	JN247557	
*G. inusitatus* var. *cystidiatus* (T)	BRNM737257	Hungary	JN247550	JN247554	JX536179
*G. iocephalus*	TENN52970	USA	DQ449984	KY019630	
*G. iodes* (T)	HGASMF01-10068	China	OM970869	OM970869	
*G. irresolutus*	SFSU-DED-8209	Sao Tome	MF100973		
*G. junquilleus* (T)	TENN55224	USA	NR_119582		
*G. kauffmanii*	DUKE230	USA	DQ450001		
*G. lanipes* (T)	BRNM670686	Spain	JX536137		JX536205
*G. loiseleurietorum*	URM90060	Sweden	KY321571	KY321572	
*G. longisterigmaticus* (T)	HMJAU60288	China	OM030282	OM033403	
***G. longistipes*** **(T)**	**HMJAU61076**	**China**	**PP646156**	**PP646168**	**PP654450**
***G. longistipes***	**HMJAU61200**	**China**	**PP646157**	**PP646169**	**PP654451**
***G. longistipes***	**HMJAU61201**	**China**	**PP646158**	**PP646170**	**PP654452**
*G. longus* (T)	HMJAU60291	China	OM030285	OM033400	
*G. luteofuscus* (T)	HMJAU61071	China	OQ597062	OQ594472	
*G. macropus*	TENN58619	Costa Rica	DQ449979		
*G. macrosporus* (T)	HMJAU60294	China	OM030266	OM033397	
*G. maculatus* (T)	HMJAU61070	China	OQ597059	OQ594469	
*G. minor* (T)	HMJAU61064	China	OQ597051	OQ594461	
*G. montagnei*	URM87715	Brazil	KT222652		
*G. neobrevipes* (T)	TENN-F-14505H1	USA	MH673477	MH673477	
*G. nubicola*	NYBG-REH-8290	Costa Rica	AF505781		
*G. obscuroides*	GB-0150514	Norway	KX958399	KX958399	
*G. ocior*	BRNM699795	Czech Republic	JX536166		JX536188
*G. omphalinoides* (T)	GDGM78318	China	MW134044	MW134730	
*G. pallipes* (T)	GDGM81513	China	MW582856	OK087327	
*G. polyphyllus*	TENN59455	USA	AY256695		
*G. pubipes*	AH26931	Spain	MZ542558	MZ542562	
*G. pygmaeus*	URM90003	Brazil	KX869966	KY088273	
*G. quercophilus*	URM90061	Sweden		KY404979	
*G. salakensis*	SFSU-AWW29	Indonesia	AY263447		
*G. schizophyllus* (T)	GDGM77165	China	MW134043	MW134729	
*G. semihirtipes*	TENN-F-07595	USA	OK376741		
*G. sepiiconicus*	SFSU-AWW126	Indonesia	AY263449		
*G. similis* (T)	BRNM766739	Korea	KP336692	KP336699	
***G. similis***	**HMJAU61204**	**China**	**PP646167**	**PP646179**	
*G. sinopolyphyllus* (T)	HMJAU60386	China	OM970872	OM970872	
***G. spadiceus*** **(T)**	**HMJAU61205**	**China**	**PP646160**	**PP646172**	**PP654454**
***G. spadiceus***	**HMJAU61206**	**China**	**PP646161**	**PP646173**	**PP654455**
***G. spadiceus***	**HMJAU61207**	**China**	**PP646162**	**PP646174**	**PP654456**
*G. spongiosus*	TENN-F-68184	USA	KY026706	KY026706	
***G. striatipileatus***	**HMJAU61199**	**China**	**PP646164**	**PP646176**	
***G. striatipileatus***	**HMJAU61074**	**China**	**PP646165**	**PP646177**	
***G. striatipileatus*** **(T)**	**HMJAU61073**	**China**	**PP646166**	**PP646178**	
*G. striatus*	HMJAU60297	China	OM030263	OM033384	
*G. strigosipes* (T)	HMAS295796	China	OM970874	OM970874	
*G. subdensilamellatus* (T)	HMJAU60997	China	ON259032	ON259042	
*G. subfasciatus* (T)	HMJAU61089	China	OQ597067	OQ594477	
*G. subpolyphyllus* (T)	HMJAU60999	China	ON259028	ON259043	
*G. subsulphureus*	TENN56321	USA	DQ449972		
*G. subsupinus*	PDD96595	New Zealand	KM975399	KM975375	
*G. talisiae* (T)	URM87730	Brazil	KT222655	KX958401	
*G. tiliicola* (T)	HMJAU60305	China	OM030275	OM033393	
*G. tomentosus* (T)	HMJAU60303	China	OM030278	OM033390	
*G. trabzonensis* (T)	KATO Fungi 3375	Turkey	KT271754		
*G. variicolor* (T)	BRNM714959	Korea	LT594121	KP348011	
***G. viridiscus*** **(T)**	**HMJAU61202**	**China**	**PP646159**	**PP646171**	**PP654453**
***G. viridiscus***	**HMJAU61203**	**China**	**PP646163**	**PP646175**	**PP654457**
*G. vitellinipes* (T)	SFSU-AWW127	Indonesia	AY263429	AY639432	
*G. westii*	FLAS-F-62668	USA	MK268238		
*Marasmiellus alnicola*	URM90019	Brazil	KY302681	KY302682	
*Marasmius aurantioferrugineus*	BRNM714752	Korea	FJ904962	MK278334	
*M. brunneospermus* (T)	KPM-NC0005011	Korea	FJ904969	FJ904951	

## Data Availability

The original contributions presented in the study are included in the article, further inquiries can be directed to the corresponding author.
